# The effectiveness of mind-body therapy and physical training in alleviating depressive symptoms in adult cancer patients: a meta-analysis

**DOI:** 10.1007/s00432-024-05813-3

**Published:** 2024-06-05

**Authors:** Yixian Zeng, Ruixin Huang, Li Zhao, Xingfei He, Shanshan Mao

**Affiliations:** 1 School of Sports Medicine and Rehabilitation, Beijing Sport University, Beijing, 100084 China; 2 School of Sports Science, Beijing Sport University, Beijing, 100084 China; 3 Wuxi Huishan District Rehabilitation Hospital, Wuxi, 214001 China

**Keywords:** Cancer, Depression, Adult, Mind-body therapy, Physical training

## Abstract

**Purpose:**

The aim of this study was to assess the effectiveness of mind-body therapy (MBT) and/or physical training in alleviating depressive symptoms among adult cancer patients through a meta-analysis.

**Methods:**

PubMed, Embase, EBSCO, Web of Science, and Cochrane Library databases were searched from up to October 21, 2023. Effect sizes, 95% confidence intervals, and other pertinent values were computed utilizing a random-effects model with Review Manager 5.3 and StataMP 14. The reporting of findings adhered to the guidelines for systematic reviews and meta-analyses. The PROSPERO registration code for this review is 4,203,477,316.

**Results:**

10 randomized controlled trials (11 datasets) involving a total of 620 participants were selected for analysis. The results demonstrated that complementary therapies, encompassing MBT and physical training, were effective in alleviating depressive symptoms in adult cancer patients (SMD= -0.47; 95%CI: -0.87, -0.08; *P* = 0.02). Subgroup analysis indicate that physical training may effectively alleviate depressive symptoms (SMD= -0.72; 95%CI: -1.31, -0.13; *P* = 0.02), demonstrating moderate effect sizes. Conversely, MBT does not seem to significantly influence depressive symptoms (*P* = 0.69).

**Conclusions:**

Complementary therapy lasting four weeks or more, incorporating physical training and MBT, has been shown to alleviate depressive symptoms in adult cancer patients. And physical training has a significant effect on depressive symptoms, while MBT has no effect. Nevertheless, given the constraints of the included studies, further research is required in the future to provide more robust evidence.

**Supplementary Information:**

The online version contains supplementary material available at 10.1007/s00432-024-05813-3.

## Introduction

Cancer stands as a profound global health challenge, ranking as the second leading cause of death worldwide(Suran 2022). Individuals with cancer are at a higher risk of developing mental illness compared to the general population(Vehling et al. 2022). Depression, in particular, is a prevalent psychological symptom among cancer patients(Jacob and Kostev 2016). The occurrence of depression can vary depending on the specific type of cancer and the stage of diagnosis(Walker et al. 2014; Ghanem et al. 2020). On average, approximately a quarter of cancer patients experience depression(Hartung et al. 2017).

The development of depression in cancer patients is associated with various risk factors, including female gender, substance abuse, history of emotional disorders and other mental illnesses, lack of social support, lower socioeconomic status, and more advanced illness(Caruso et al. 2017b; Wang et al. 2020a). Depression can have several negative impacts on cancer patients, such as alleviated quality of life, treatment non-adherence, less optimistic prognosis, and increased mortality rates (Caruso et al. 2017a, b; Wang et al. 2020a, b). Therefore, it is crucial to address and alleviate depressive symptoms in cancer patients to alleviate overall well-being and outcomes.

Several studies have demonstrated the efficacy of psychological and psychopharmacological therapies in treating depression(Driessen et al. 2010; Fournier et al. 2010). Research suggests that exercise can impact depression through mechanisms similar to those of antidepressant medication. Combining exercise with medication may have an additive effect in alleviating depressive symptoms(Micheli et al. 2018). Exercise offers a viable option for reducing depressive symptoms. The association between exercise and depression can be attributed to various mechanisms(Moon et al. 2012; Kandola et al. 2019; Smith and Merwin 2021), including the release of anti-inflammatory cytokines during exercise, which aids in alleviating inflammation—a primary biological factor linked to depression.

Previous research has demonstrated the beneficial effects of exercise on depression. For instance, a meta-analysis conducted by Morres in 2019 revealed that engaging in 45 min of moderate-intensity aerobic training (AT), three times per week, over a period of 9.2 weeks, led to a significant improvement in depression levels among adult patients diagnosed with major depression(Morres et al. 2019). Furthermore, in a subsequent study conducted by Morres in 2022, it was found that exercise, particularly when incorporating ≥ 150 min of moderate-intensity AT, had a notable antidepressant effect in women experiencing perinatal depressive symptoms(Morres et al. 2022).

However, it is important to note the existing controversy regarding the efficacy of exercise in alleviating depression symptoms among individuals with cancer. Schmitz et al. suggest that engaging in moderate-intensity aerobic training three times per week for 30 min each session is recommended. Additionally, they suggest that the combination of AT and resistance training (RT) does not yield benefits for cancer-related depression(Schmitz et al. 2021). However, the American College of Sports Medicine (ACSM) guidelines recommend AT three times weekly for at least 12 weeks, or a blend of AT and RT twice weekly for 6 to 12 weeks, as effective approaches to alleviate depressive symptoms in cancer survivors(Marconcin et al. 2022). Furthermore, a meta-analysis has demonstrated that physical training, encompassing RT and AT, can significantly alleviate depressive symptoms in cancer patients(Law et al. 2023). The conflicting outcomes in these studies highlight the absence of a consensus regarding the efficacy of physical training in addressing depression among cancer patients.

Certain cancer patients may face obstacles to engaging in exercise(Courneya et al. 2008). Gentler forms of exercise, such as yoga, have been found to help motivate this group to engage in regular exercise(Buffart et al. 2012). Mind-body therapies (MBTs), which encompass relaxation, hypnosis, yoga, meditation, Tai Chi, and Qigong, are considered complementary therapies for cancer(Deleemans et al. 2023), aligning with the MBTs considered in our study. Previous studies have shown that MBTs have a positive impact on reducing depression(Duan et al. 2020). It is worth noting that the scope of MBTs analyzed in Duan’s article includes dance, which differs from the definition of MBT mentioned in our research. Additionally, some of the participants in the mentioned article had lower depression scores at baseline, which may limit the potential for further improvement or change during the intervention. This phenomenon could be attributed to a baseline effect(Altman and Bland 1999).

Not all recent studies have provided evidence supporting the effectiveness of MBTs in alleviating depressive symptoms in cancer patients. For example, Eggers et al. conducted a spiritual meditation intervention for pancreatic cancer survivors and found no significant change in depression scores over a 12-month observation period (Eggers et al. 2023). Similarly, The study conducted by Wen et al. involved a 12-week Baduanjin intervention, which is a form of Qigong, for nasopharyngeal carcinoma patients and observed no noteworthy distinction in depressive symptoms between the control and intervention groups by week 12 of their study(Wen et al. 2023). It means that the effectiveness of MBTs in relieving depressive symptoms in cancer patients remains uncertain.

A study conducted by Knoerl et al. demonstrated that both preoperative physical exercise and MBT can effectively alleviate perioperative depression levels in early-stage cancer patients. However, the study did not identify a statistically significant difference in the effectiveness between these two interventions. Additionally, the absence of a control group in the study design limits the ability to draw definitive conclusions regarding the efficacy of physical exercise and MBT(Knoerl et al. 2022).

Historically, both physical training and MBTs have been explored as complementary approaches for managing cancer-related symptoms. Nevertheless, their specific effectiveness in addressing cancer-related depression needs to be further confirmed. If MBTs and physical training are deemed viable options for conservative treatments targeting cancer-related depression, it could expand the range of available choices for personalized exercise programs tailored to adult cancer patients.

In summary, this meta-analysis aims to compare the efficacy of MBTs (including meditation, hypnosis, relaxation, yoga, Tai Chi, and Qigong) and physical training (such as RT and AT) in addressing depression among adult cancer patients. The goal is to provide valuable health information for adult cancer patients with depression and aid clinical decision-making processes.

## Materials and methods

### Search strategy

The meta-analysis adhered to the procedures outlined in the Cochrane Handbook for Systematic Reviews of Interventions and was reported in compliance with the Preferred Reporting Items for Systematic Reviews and Meta-Analyses (PRISMA) guidelines. The review protocol for this study was registered in the PROSPERO database with the registration number CRD 4,203,477,316.

This search was conducted across multiple databases, including PubMed, Embase, EBSCO, and Web of Science, and the Cochrane Library. The search covered articles published between January 1, 2000, and October 21, 2023. The search strategy employed various terms related to “cancer,” “depression,” “adult,” “mind-body therapy,” “Tai Chi,” “Qigong,” “meditation,” “hypnosis,” “relaxation therapy,” “yoga,” “physical exercise,” “aerobic exercise,” and “randomized controlled trial.” Furthermore, the references of the selected articles underwent scrutiny to uncover pertinent studies.

### Eligibility criteria

Two reviewers, YXZ and RXH, meticulously scrutinized the titles and abstracts of the retrieved literature to pinpoint relevant studies for the research. Only articles with titles and abstracts directly pertinent to the aim of the review were advanced for further full-text screening. The inclusion criteria for the articles were as follows: (1) participants: adult patients diagnosed with any type of cancer undergoing active treatment or in recovery; (2) intervention: various MBTs such as Tai Chi, Qigong, yoga, meditation, hypnosis, and relaxation, as well as physical training including aerobic and resistance training, either alone or in combination with other specified interventions; (3) control group: usual care, health education, chemotherapy, or wait-list control group; (4) outcome: assessment of the severity or level of depressive symptoms using scales such as the Hospital Anxiety and Depression Scale (HADS), the Beck Depression Inventory (BDI), or the Beck Depression Inventory-II (BDI-II); and (5) study: only randomized controlled trials (RCTs) were considered for inclusion.

The inclusion criteria were limited to articles published in English. Articles meeting any of the following conditions were excluded: (1) patients with non-significant baseline levels of depression based on the assessment tool used; (2) interventions not specified in this review; (3) lack of a control group; (4) absence of pre- or post-trial data; and (5) non-RCTs.

### Data extraction

Data extraction was independently carried out by the two reviewers (YXZ and RXH), encompassing the following details: the first author’s name and publication year, along with characteristics of the study population such as age and gender, baseline depression levels, total and group sample sizes, intervention details (type, frequency, duration, etc.), control group information, depression inventory used, and outcomes (mean and standard deviation of depression levels in each group after the intervention). In cases of disagreement between the two reviewers, a third reviewer (SSM) was consulted to resolve any disputes.

Efforts were made to address missing data by contacting the authors in order to obtain the necessary information. Unfortunately, despite these attempts, no additional data was received.

### Quality of assessment

The Cochrane risk-of-bias tool (RoB) was used to assess bias risk in the included RCTs. It evaluated various domains including the randomization process (allocation sequence and concealment), deviations from intended interventions, missing outcome data, measurement of the outcome, and selection of reported results. Each domain was categorized as having a “low risk of bias,” “some concerns,” or “high risk of bias” based on the assessment.

Funnel plots were employed to investigate potential publication bias across all outcome measures in the study series. Furthermore, sensitivity analysis was conducted to evaluate the influence of individual studies on the meta-analysis estimates. The GRADE Evidence Rating Scale was employed to rate the trial results, providing an assessment of the overall quality of evidence(Cohen 1988).

### Data analysis

A meta-analysis was conducted to assess changes in scores of the depressive symptom scale before and after the intervention in cancer patients. Mean differences and standard deviations were extracted from the intervention and control groups to estimate the effect size for alleviating depressive symptoms. Given the inconsistent utilization of depression rating scales across studies, standardized mean differences (SMDs) with 95% confidence intervals were employed to measure continuous outcomes.

Heterogeneity between the included studies was evaluated using I^2^ and Chi^2^ tests, with estimated *p*-values, performed in Review Manager 5.3 and Stata/MP 14. Heterogeneity was considered present when the *p*-value was < 0.1, and I^2^>50%(Cochrane Training 2022). Sensitivity analysis and subgroup analysis were conducted to identify potential sources of heterogeneity whenever detected. Despite these efforts, heterogeneity persisted, leading to the utilization of a random-effects model.

Subgroup analyses and meta-regression were utilized to explore the impact of scale type, intervention duration, and intervention type on exercise for depressive symptoms in cancer survivors. Specifically, meta-regression aimed to compare the effects of the two intervention types on depression symptoms in adult cancer survivors. Sensitivity analyses were conducted to assess the robustness of the findings.

Funnel plots and the Egger’s test, using Review Manager 5.3 software, were employed to evaluate publication bias. These methods were utilized to ensure the integrity and reliability of the study’s conclusions.

## Results

### Study selection

**Fig. 1:**
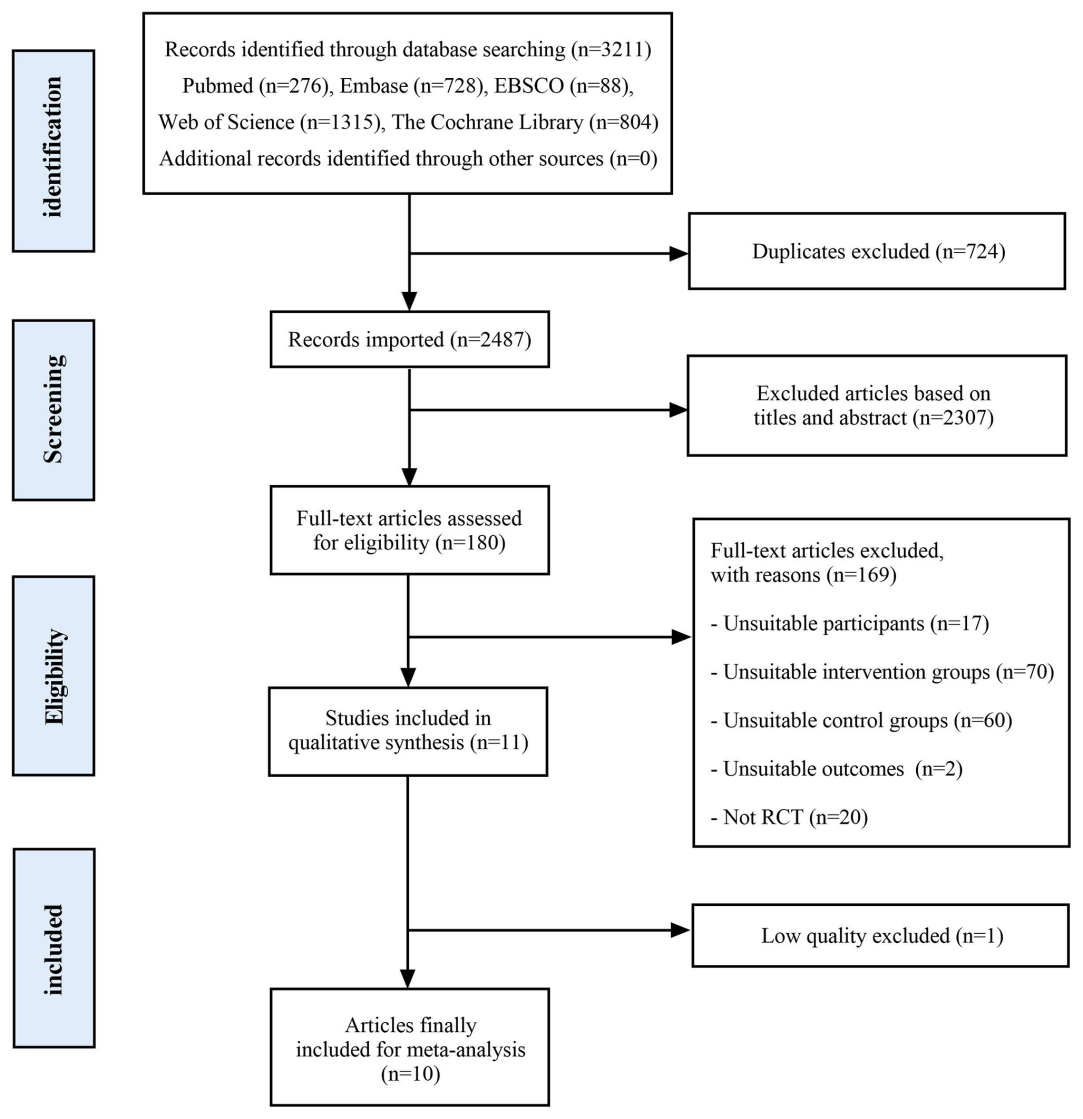
The article selection process flow diagram in accordance with the PRISMA

A comprehensive search of databases yielded a total of 3,211 articles. After removing duplicates and conducting a thorough review of the full-text articles, 11 RCTs met the eligibility criteria for inclusion. However, one of the RCTs(Danhauer et al. 2009) was deemed to have poor quality based on the RoB assessment. Consequently, this particular RCT was excluded, resulting in a final selection of 10 RCTs that were included in the meta-analysis (Fig. 1).

### Characteristics of studies

The details were summarized in Table 1.

**Table 1:** Characteristics of the included studies

First author Year	Sample size (randomized/ analyzed/ dropout)	Population (Stage)	Age (years old) Gender (*n*)	Intervention	Comparison	Measure time	Outcome: Measurement	The severity level of depression at pre-intervention (Formal diagnosis of depression)
Ho (2020)	112/112/0	CRC (I-IV)	IG: 66.6 ± 9.5, 40 M, 16 F CG: 64.9 ± 9.4, 30 M, 26 F	***Physical training*** **physical activity**: moderate to vigorous intensity, 30–60 min/day, 5 days/week, 12 months	Usual care	Immediately post-intervention	Depressive symptom: HADS	Moderate (NR)
Cornette (2015)	44/19/25	BC (I-III)	IG: 52 (range = 37 to 73) CG: 49 (range = 37 to 68) only F	***Physical training*** **mixed exercise**: aerobics: 20–40 min/time, resistance: two sets of 8–12 repetitions/time, 3 times/week, 27 weeks	Usual care	Immediately post-intervention	Depressive symptom: HADS	Moderate to severe (NR)
Mutrie (2007)	203/174/29	BC (0-III)	IG: 51.3 ± 10.3 CG: 51.8 ± 8.7 only F	***Physical training*** **mixed exercise**: aerobics, strength, etc., 50–75% HR_max_, 45 min, 3 times/week, 12 weeks	Usual care	Immediately post-intervention	Depressive symptom: BDI	Potential subclinical (NR)
Mostafaei (2021)	60/60/0	BC (0-III)	IG: 48.46 ± 5.72 CG: 49.60 ± 7.48 only F	***Physical training*** **mixed exercise**: dynamic, resistance and balance, 20-30-min, 3 sessions/week, 6 weeks	Usual care	Immediately post-intervention	Depressive symptom: BDI-II	Mild (NR)
Rehman (2023)	40/40/0	NSCLC (I -II)	IG: 48.1 ± 4.0, 11 M, 9 F CG: 48.3 ± 3.8, 13 M, 7 F	***Physical training*** **PR plus aerobic exercise**: 40–60% intensity, 30 min/day, 5days/week, 4weeks	Only PR	Two weeks post-intervention	Depressive symptom: HADS	Moderate (NR)
Eisenhut (2022)	29/27/2	HGG (III-IV)	IG: 49.1 ± 13.14, CG: 53.0 ± 10.78 Total (3 groups): 13 M, 16 F	***Physical training*** **endurance training**: Borg scale 11–14, 35–45 min, 2 sessions/week, 6 weeks	Advice	Immediately post-intervention	Depressive symptom: BDI	Potential subclinical (NR)
Eisenhut (2022)	29/27/2	HGG (III-IV)	IG: 54.6 ± 13.45, CG: 53.0 ± 10.78 Total (3 groups): 13 M, 16 F	***Physical training*** **strength training**: 3–5 series of 10–15 repetitions, Borg scale 11–15, 35–45 min, 2 sessions/week 6 weeks.	Advice	Immediately post-intervention	Depressive symptom: BDI	Potential subclinical (NR)
Bower (2012)	31/31/0	BC (0-II)	IG: 54.4 ± 5.7 CG: 53.3 ± 4.9 only F	***MBT*** **yoga**: 90 min, twice/week, 12 weeks	Education	Immediately post-intervention	Depressive symptom: BDI-II	Mild (NR)
Cramer et al. (2015)	40/40/0	BC (I-III)	IG: 48.3 ± 4.8 CG: 50.0 ± 6.7 only F	***MBT*** **yoga**: 90 min/week, 12 weeks	Usual care	Immediately post-intervention	Depressive symptom: HADS	Potential subclinical (NR)
Larkey (2015)	101/81/20	BC (0-III)	IG: 57.7 ± 8.94 CG: 59.8 ± 8.93 only F	***MBT*** **Tai Chi or Qigong**: 30 min/day, 5 day/week, 12 weeks	Sham	Immediately post-intervention	Depressive symptom: BDI	Potential subclinical (NR)
Eyigor (2018)	42/36/6	BC (NR)	IG:52.3 ± 9.5 CG:51.5 ± 7.3 only F	***MBT*** **yoga**: 60 min, 2 days/week, 10 weeks.	Usual care	Immediately post-intervention	Depressive symptom: BDI	Potential subclinical (NR)

**Abbreviations**: IG, intervention group; CG, control group; M, males; F, females; MBT, mind-body therapy; CRC, colorectal cancer; BC, breast cancer; PR, pulmonary rehabilitation; NSCLC, non-small cell lung cancer; HGG, high-grade glioma; HADS: hospital anxiety and depression scale; BDI, Beck depression inventory. NR, this characteristic of cancer patients was not referred in the study.

### Participants

The study comprised a total of 620 adult cancer patients (comprising cancer stages 0- IV), with the mean age ranging from 48 to 67 years. Seven studies (70.0%) exclusively enrolled female participants (Mutrie et al. 2007; Bower et al. 2012; Larkey et al. 2015; Cramer et al. 2015; Cornette et al. 2016; Eyigor et al. 2018; Mostafaei et al. 2021), while three studies (30.0%) had a mixed population(Ho et al. 2020; Eisenhut et al. 2022; Rehman et al. 2023). The selected RCTs involved patients with a variety of cancer types, such as breast cancer (*n* = 443), colorectal cancer (*n* = 112), non-small cell lung cancer (*n* = 40), and high-grade glioma (*n* = 27). Based on the assessment scales used in the included articles, the patients exhibited depressive symptoms at baseline that met clinical criteria. Although the formal diagnosis of depression was not explicitly mentioned, six trials indicated the presence of potentially subclinical depressive states based on the assessment tools utilized, two trials indicated mild depression, two trials indicated severe depression, and one trial indicated moderate to severe depression.

### Interventions

The interventions in this study can be categorized into two main groups: MBT and physical training. Among the four studies that administered MBT, three utilized yoga as the intervention (Bower et al. 2012; Cramer et al. 2015; Eyigor et al. 2018), while one study employed Qigong or Tai Chi(Larkey et al. 2015). On the other hand, physical training was implemented in six trials, with three studies using mixed exercises(Mutrie et al. 2007; Cornette et al. 2016; Mostafaei et al. 2021), one focusing on increasing physical activity (Ho et al. 2020), one combining pulmonary rehabilitation with aerobics (Rehman et al. 2023), and one involving aerobic or strength training (this study had two experimental groups) (Eisenhut et al. 2022).

### Outcome measures

Depressive symptoms were assessed using different scales across the included studies. The HADS was used in four studies(Cramer et al. 2015; Cornette et al. 2016; Ho et al. 2020; Rehman et al. 2023), the BDI was employed in four studies(Mutrie et al. 2007; Larkey et al. 2015; Eyigor et al. 2018; Eisenhut et al. 2022), and the BDI-II was utilized in two studies(Bower et al. 2012; Mostafaei et al. 2021). All outcome measures are continuous data. To account for variations in score ranges and scoring criteria among the different scales, SMDs will be calculated for the outcome analysis.

### Risk of bias

**Fig. 2:**
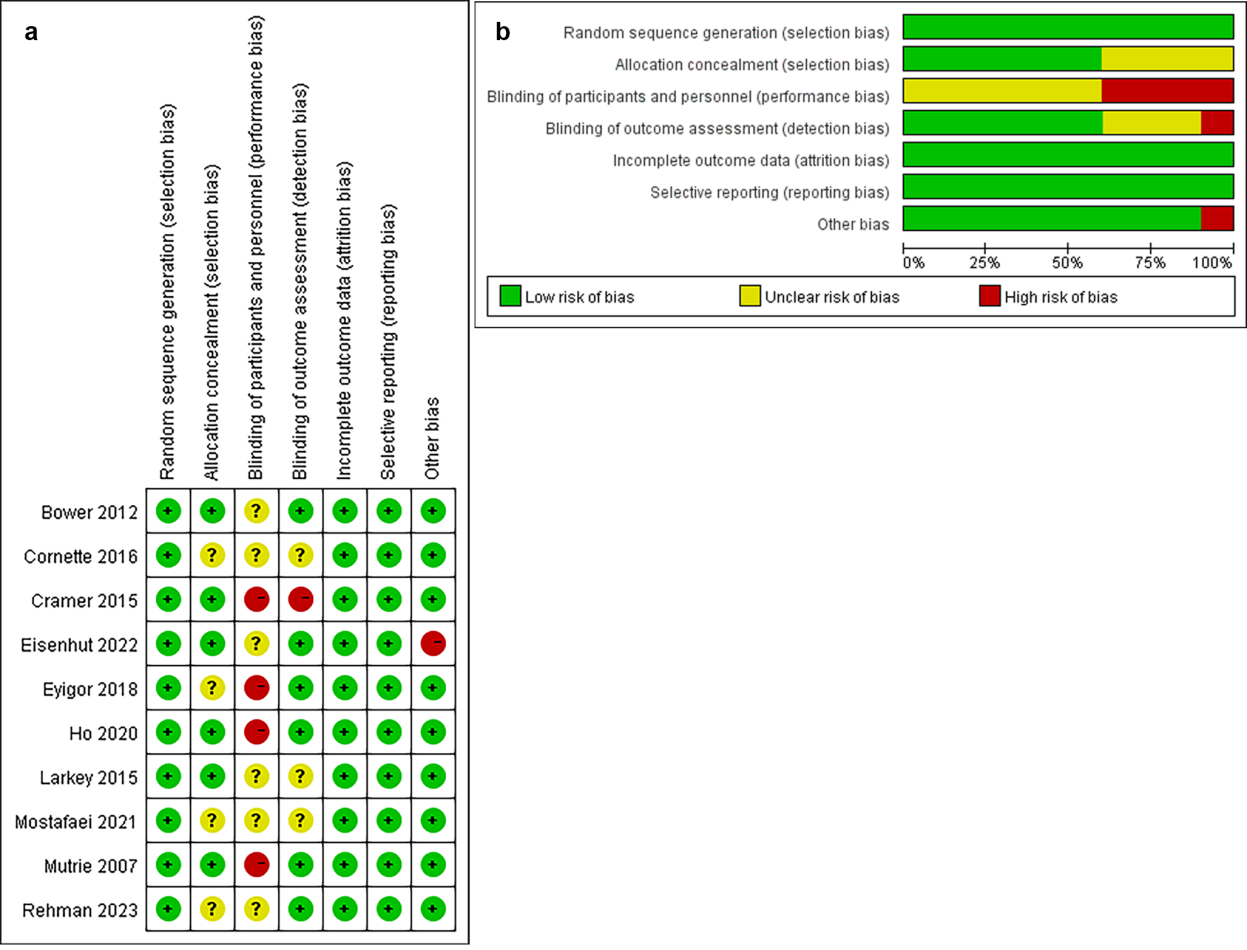
The authors’ evaluations of each item for each study included. **(A)** Risk of bias summary; **(B)** Risk of bias graph

According to the RoB assessment, it was observed that due to the inherent nature of the exercise interventions, participants in all included studies were not blinded to the intervention, indicating an unclear or high risk of performance bias. Additionally, some studies showed uncertainty regarding bias in terms of allocation concealment and blinding of outcome assessment. Moreover, one study was flagged for ‘other bias’ due to its small sample size (Fig. 2).

### Publication bias

A funnel plot (Supplementary Fig. 1) and Egger’s test (Supplementary Fig. 2) were conducted on the 10 studies, which consisted of 11 datasets. The funnel plot demonstrated a symmetric distribution of the data points, implying no significant publication bias. Moreover, the results of Egger’s test indicated *P* = 0.124>0.05. This finding further supports the conclusion that there was no substantial publication bias among the included studies.

### Quality of evidence

The level of certainty regarding the impact of exercise in reducing depressive symptoms among adults with cancer was determined to be low (Supplementary Fig. 3).

### Data synthesis

#### Meta-analysis

**Fig. 3:**
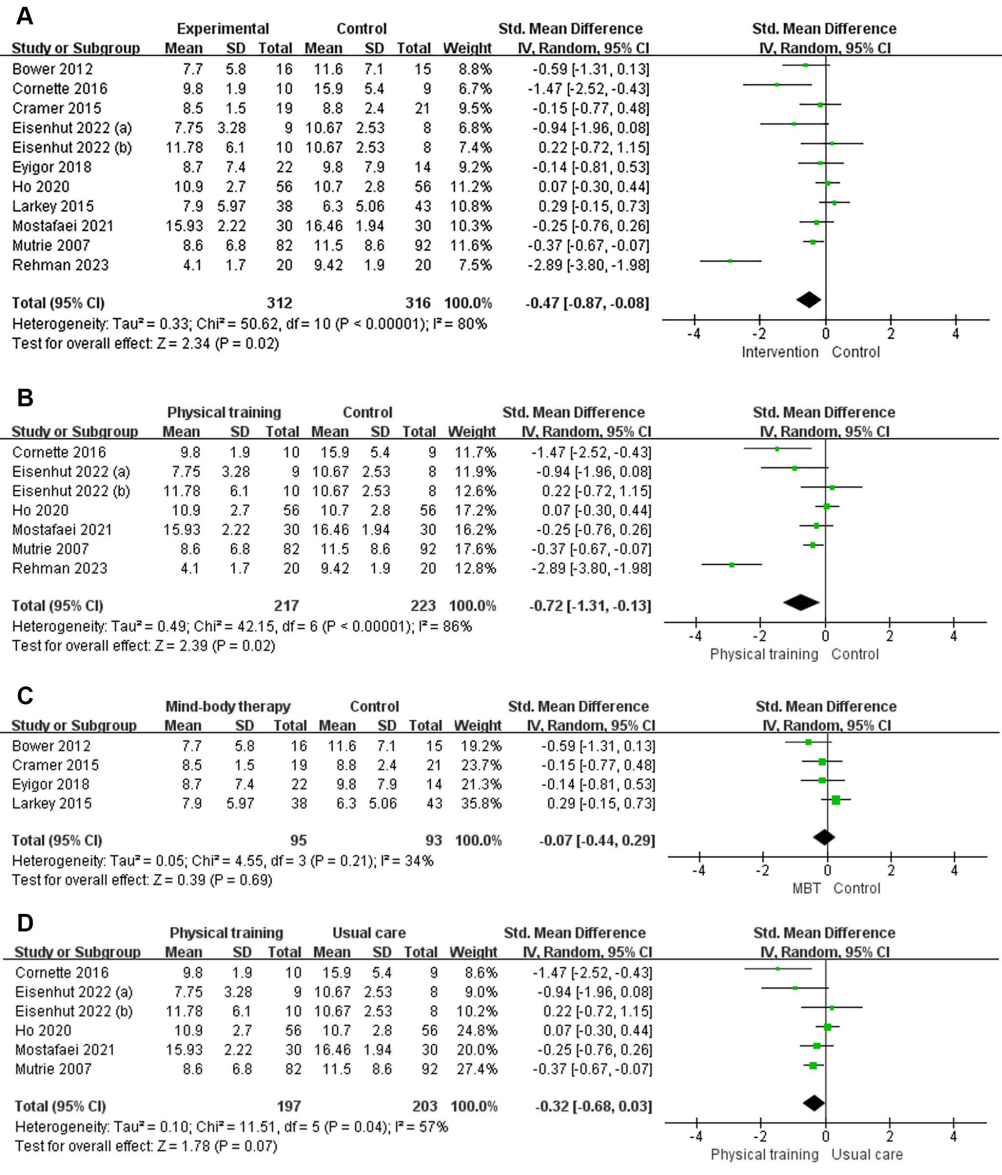
Forest plots. **(A)** Forest plot of interventions (including physical training and MBT) on depressive symptoms in cancer patients; **(B)** Forest plot of physical training on depressive symptoms in cancer patients; **(C)** Forest plot of MBT on depressive symptoms in cancer patients; **(D)** Forest plot of physical training vs. usual care on depressive symptoms in cancer patients

In the meta-analysis exploring interventions (including physical training and MBT) for depression symptoms, 10 studies comprising 11 datasets were incorporated. The findings uncovered notable heterogeneity, with an I² statistic of 80% (indicating substantial variability beyond chance, given I² > 50%) and a *p*-value of less than 0.001. As a result, a random effects model was employed (Fig. 3).

The random-effects meta-analysis unveiled a significant reduction in depression levels within the intervention group, which included both physical training and MBT, compared to the control group. Specifically, the intervention group exhibited a mean difference of -0.47 (95% CI: -0.87, -0.08) in contrast to the control group, with a *p*-value of 0.02, indicating statistical significance. The observed effect sizes were in the small range according to Cohen’s classification, where 0.2 ≤ small < 0.5, 0.5 ≤ medium < 0.8, and 0.8 ≤ large effect sizes(Cohen 1988).

#### Sensitivity analysis

A sensitivity analysis was performed on the 10 studies included in this study using a one-by-one exclusion method. This analysis aimed to assess the influence of each individual study on the overall effect and the robustness of the results. The results of the sensitivity analysis are presented in the accompanying illustration (Supplementary Fig. 4).

Excluding the study by Rehman (2023) from the analysis led to the combined effect size losing statistical significance. Therefore, the findings of the study are not robust. Furthermore, the Ho (2020) physical training intervention was not subject to supervision, and a sensitivity analysis was conducted once more following its exclusion. The findings indicate that Rehman (2023) continues to be the source of results that lack robustness (Supplementary Fig. 5). The researchers will carefully examine the differences between this particular study and others, considering clinical and methodological factors, in order to identify the specific factors contributing to the observed heterogeneity.

#### Subgroup analyses

##### Intervention type

A total of 10 studies (comprising 11 datasets) were included in the analysis, divided into two subgroups based on the intervention type: MBT and physical training. Among the included studies, four focused on MBT interventions(Bower et al. 2012; Larkey et al. 2015; Cramer et al. 2015; Eyigor et al. 2018) and 6 studies performed physical training(Mutrie et al. 2007; Cornette et al. 2016; Ho et al. 2020; Mostafaei et al. 2021; Eisenhut et al. 2022; Rehman et al. 2023).

The results indicated that physical training significantly alleviated depression symptoms in cancer patients, as evidenced by a SMD of -0.72 (95%CI: -1.31, -0.13), Z = 2.39, and *P* = 0.02 < 0.05, demonstrating statistical significance (Fig. 3B). Moreover, the effect sizes observed in the physical training subgroup was moderate.

In contrast, there was no evidence supporting the effectiveness of MBT in alleviating depression symptoms in cancer patients, with Z = 0.39 and *P* > 0.05(Fig. 3C).

Furthermore, physical training did not result in the alleviation of depressive symptoms in adult cancer patients compared with usual care, with Z = 1.78 and *P* = 0.07(Fig. 3D).

### Meta-regression

In addition to the intervention type, we sought to ascertain whether the intervention duration, the type of scale employed, and the cancer type being treated were sources of heterogeneity in the articles. Meta-regression analyses were conducted using intervention duration (T = 0.39, *P* = 0.705 > 0.05), scale type (T = 0.74, *P* = 0.478 > 0.05), and cancer type (T = -0.81, *P* = 0.440 > 0.05) as covariates. However, the results did not reach statistical significance (Supplementary Table 1).

## Discussion

In this meta-analysis, systematically reviewing studies published since 2000 to assess the impact of physical training and MBTs on reducing depressive symptoms in adult cancer survivors. The aim was also to investigate the comparative effectiveness of MBTs and physical training. In this meta-analysis, 10 RCTs (11 datasets) were included, involving a total of 620 cancer survivors. This finding demonstrates that complementary therapies, including physical training and MBTs, exhibit a significant positive effect in mitigating depressive symptoms among cancer patients (*P* = 0.02). The observed effect size is small (d = 0.47), indicating limited practical relevance and potential utility in clinical practice(Sullivan and Feinn 2012). Subgroup analyses were performed to identify potential moderators, and the results showed that physical training significantly alleviated depressive symptoms in cancer survivors with moderate effects(d= -0.72), consistent with previous research findings (Craft et al. 2012). However, Law’s study concluded that physical training had a small effect size (d= -0.16). This may be due to the fact that their subjects’ baseline depression scores were within the normal range(Law et al. 2023). In contrast, our research included adult cancer patients who had been diagnosed with depression. It is noteworthy that physical training demonstrated a moderate effect size in alleviating depressive symptoms in adult cancer patients when the control group included usual care and other measures. In order to ascertain the impact of physical training versus usual care, a subgroup analysis was conducted. Surprisingly, no evidence emerged to support the superiority of physical training over the usual care. Nevertheless, it is not sufficient to definitively conclude that physical training is ineffective in addressing depression associated with cancer, necessitating further research in the future.

The results of the study indicate that PT is efficacious, whereas MBT is not. However Duan’s study(Duan et al. 2020) indicates that MBT has the potential to significantly alleviate depressive symptoms (SMD = -0.21; 95% CI: -0.42, -0.01; *P* = 0.04), which is inconsistent with the findings of the present study. The following possible explanations account for this phenomenon:

Study design of included MBT articles: (1) Selection criteria: Our research specifically focused on participants who met the criteria for depression at baseline, which resulted in a limited number of eligible MBT studies and a smaller sample size of participants. This restricted pool of studies may have influenced the overall findings and statistical power. Nevertheless, other pertinent studies(Duan et al. 2020) did not necessitate that participants met diagnostic criteria for depression prior to intervention; (2) Lack of clarity in MBT parameters: Some studies included in the analysis did not clearly define the intensity, frequency, and duration of MBT interventions. The lack of standardized and well-defined exercise protocol in MBT interventions may have contributed to the unsatisfactory efficacy; (3) Variability in study design: The included articles exhibited variability in the duration of interventions (Bower et al. 2012) and the instructors delivering the exercise programs (Larkey et al. 2015) between the control and intervention groups.

The differences in the underlying mechanisms: (1) The exercise intensity of MBT may be comparatively lower than that of physical training. Physical training often entails higher levels of intensity, encompassing aerobic exercises and moderate strength training, which facilitate the promotion of metabolic activity, enhancement of cardiorespiratory function, and release of endogenous chemicals that can positively influence depressive symptoms. Furthermore, high-intensity exercise can elicit the body’s stress response and augment its adaptability, which holds particular significance for individuals battling cancer. Conversely, MBT emphasizes psychological interventions that may not yield equivalent physiological advantages to those achieved through high-intensity physical training. (2) MBT and physical training may exert their effects on alleviating depressive symptoms through distinct biological pathways. MBT primarily modulates the psychological state by regulating neuroendocrine, immune, and autonomic nervous systems (Streeter et al. 2010). In contrast, physical training not only operates through these mechanisms but also enhances the release of neurotransmitters, such as dopamine and serotonin, in the brain, leading to an antidepressant effect(Meeusen and De Meirleir 1995). Additionally, physical training has been shown to regulate the inflammatory response by reducing levels of inflammatory markers like C-reactive protein and cytokines, which has been associated with a reduction in depression (Fedewa et al. 2017). The impact of MBT on physiological processes is relatively limited and may not directly influence the inflammatory response.

Our results indicate that physical training could be beneficial in alleviating depressive symptoms among cancer patients, while MBT does not yield the same effect. However, it is crucial to acknowledge the presence of heterogeneity among the selected literature, as indicated by I^2^ = 80% >50% and *P* = 0.0 < 0.1 from the Q-test involving the 10 studies included in this analysis.

Subgroup analysis and meta-regression analysis was performed to explore potential sources of heterogeneity, including intervention type, intervention duration, scale type, and cancer type. However, none of these factors were identified as significant contributors to the observed heterogeneity. Additional methodological scrutiny was conducted in this study. Furthermore, sensitivity analysis raised concerns about the study conducted by Rehman (2023), which may have weakened the overall conclusions. In Rehman’s study, the intervention group received pulmonary rehabilitation in addition to exercise. It is possible that the improved quality of life may have also eased patients’ depression. Consequently, the effects of exercise may be overestimated, and caution should be exercised when interpreting the results. Furthermore, the absence of publication bias ensures the impartiality of our findings.

### Limitations

This paper has several limitations. Firstly, the limited number of articles included in the analysis restricted the possibility of conducting more detailed subgroup analyses, such as exploring different forms of exercise within the category of physical training. However, this scarcity of the studies can be attributed to our focus on a population had been diagnosed with depression. Future studies are recommended to widen the search across a broader range of databases or refine the search strategy to increase the pool of available articles for analysis. Furthermore, additional research with rigorous quality is required. Secondly, the included articles exhibited a considerable degree of heterogeneity, and no source of heterogeneity was identified by meta-regression and subgroup analyses. And all included studies had an unclear or high risk of bias in performance bias due to the inherent nature of exercise. Thirdly, the broad content of MBT may contribute to the heterogeneity observed across the articles. As researchers have not yet reached a consensus on the specific components of MBT, further refinement is necessary in future studies. Fourthly, female breast cancer patients were disproportionately represented, with 7 out of 10 studies (11 datasets) focused on this population. Therefore, future research should aim to investigate gender-specific responses.

## Conclusions

Complementary therapies, including physical training and MBT, are an efficacious approach to alleviating depressive symptoms in adult cancer patients. And more than four weeks of physical training can alleviate depressive symptoms in adult cancer patients, whereas MBT does not. However, further research is required in order to validate the findings to a greater extent.

### Electronic supplementary material

Below is the link to the electronic supplementary material.

Supplementary Material 1 Supplementary Material 1

Supplementary Material 2 Supplementary Material 2

Supplementary Material 3 Supplementary Material 3

Supplementary Material 4 Supplementary Material 4

Supplementary Material 5 Supplementary Material 5

Supplementary Material 6 Supplementary Material 6

## Data Availability

Data is provided within the manuscript or supplementary information files.
