# Supplementary figures and images for: The effectiveness of mind-body therapy and physical training in alleviating depressive symptoms in adult cancer patients: a meta-analysis
Source: J Cancer Res Clin Oncol. 2024 Jun 5;150(6):289. doi: 10.1007/s00432-024-05813-3 (PMC11153279; doi:10.1007/s00432-024-05813-3)

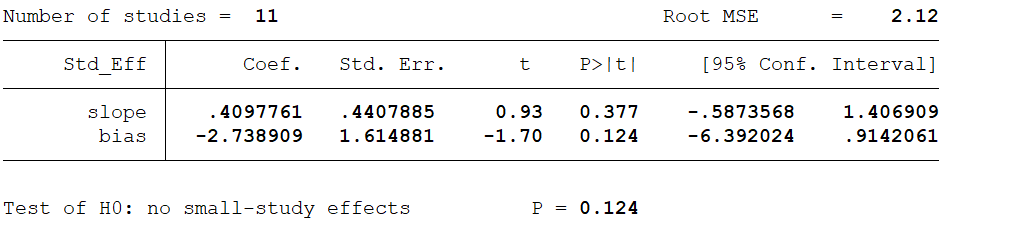

Supplement: Supplementary file 1 — Supplementary Material 1 [file 432_2024_5813_MOESM1_ESM.png]

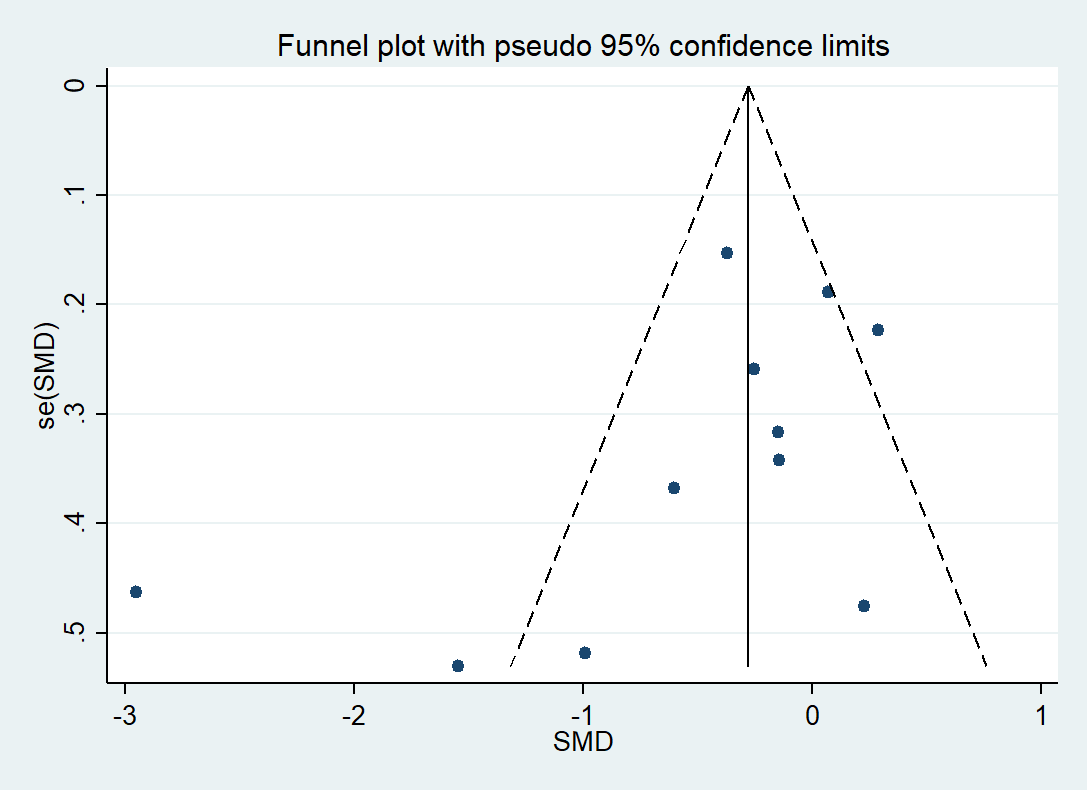

Supplement: Supplementary file 2 — Supplementary Material 2 [file 432_2024_5813_MOESM2_ESM.png]

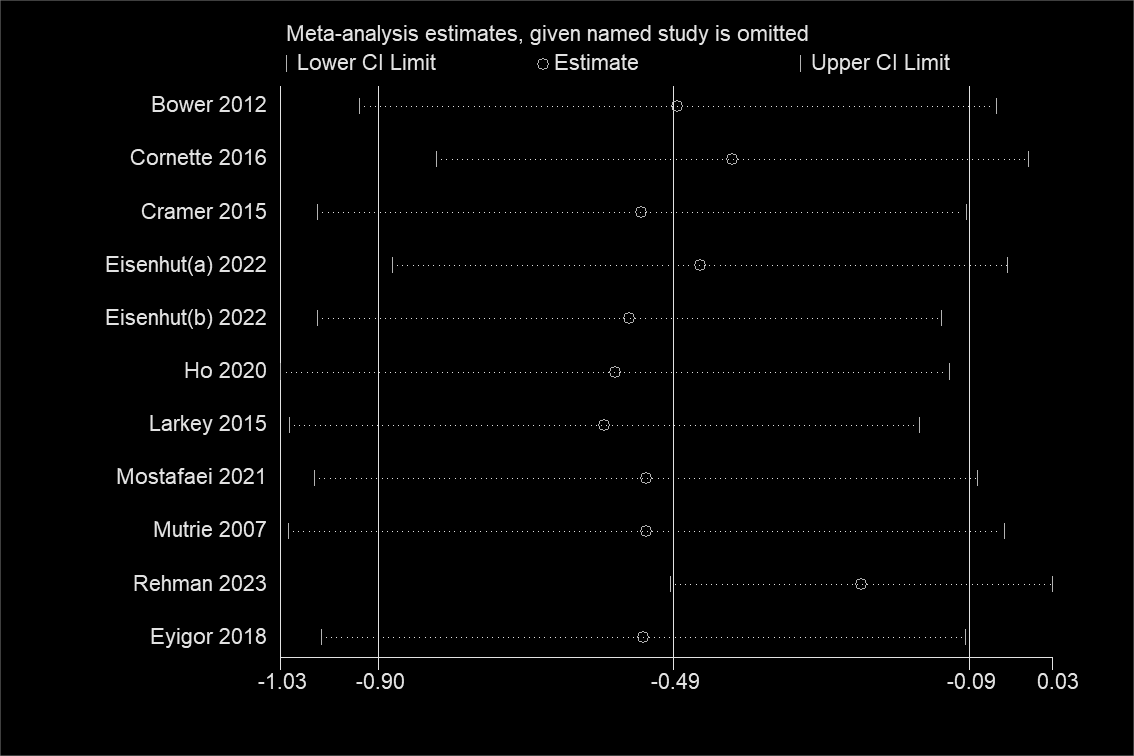

Supplement: Supplementary file 3 — Supplementary Material 3 [file 432_2024_5813_MOESM3_ESM.png]

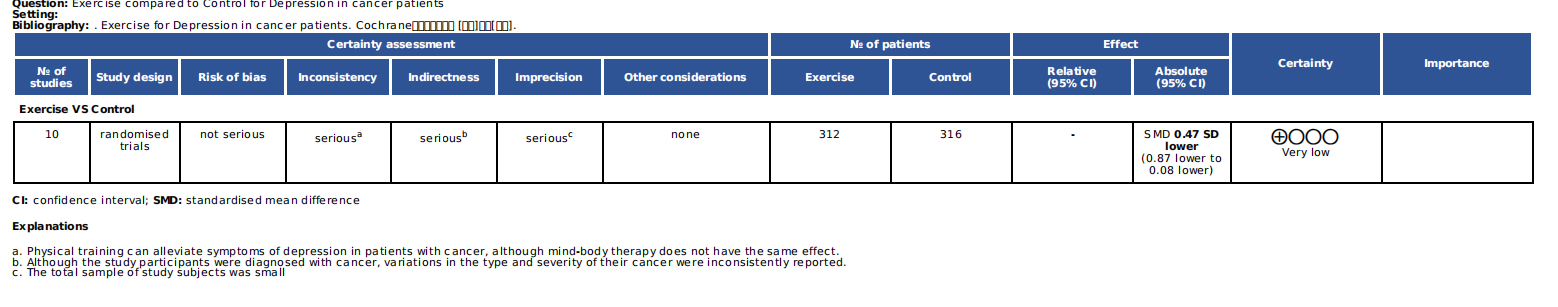

Supplement: Supplementary file 4 — Supplementary Material 4 [file 432_2024_5813_MOESM4_ESM.png]

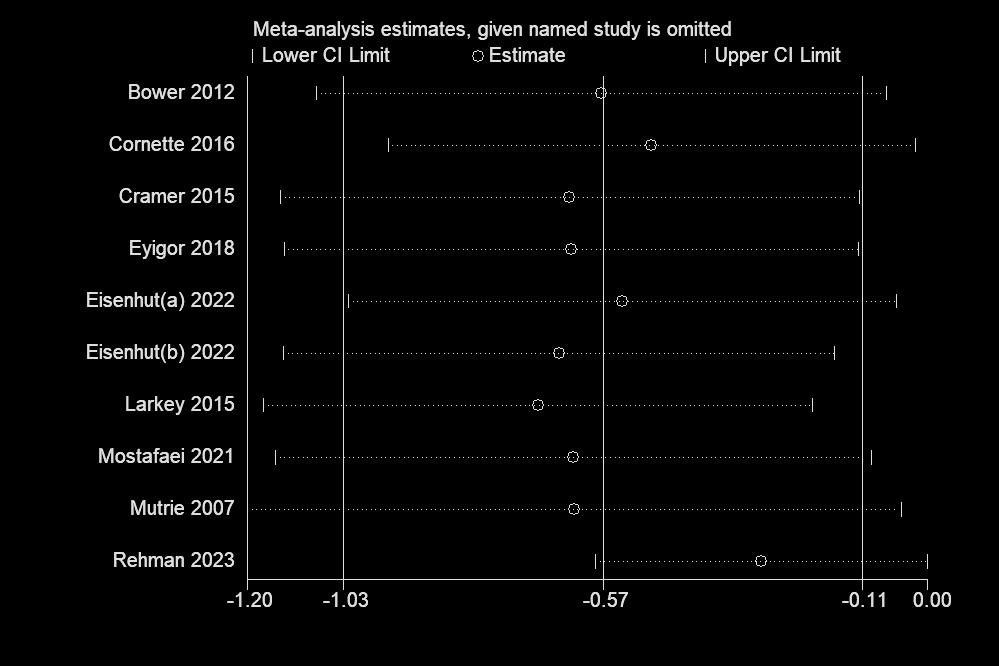

Supplement: Supplementary file 5 — Supplementary Material 5 [file 432_2024_5813_MOESM5_ESM.png]

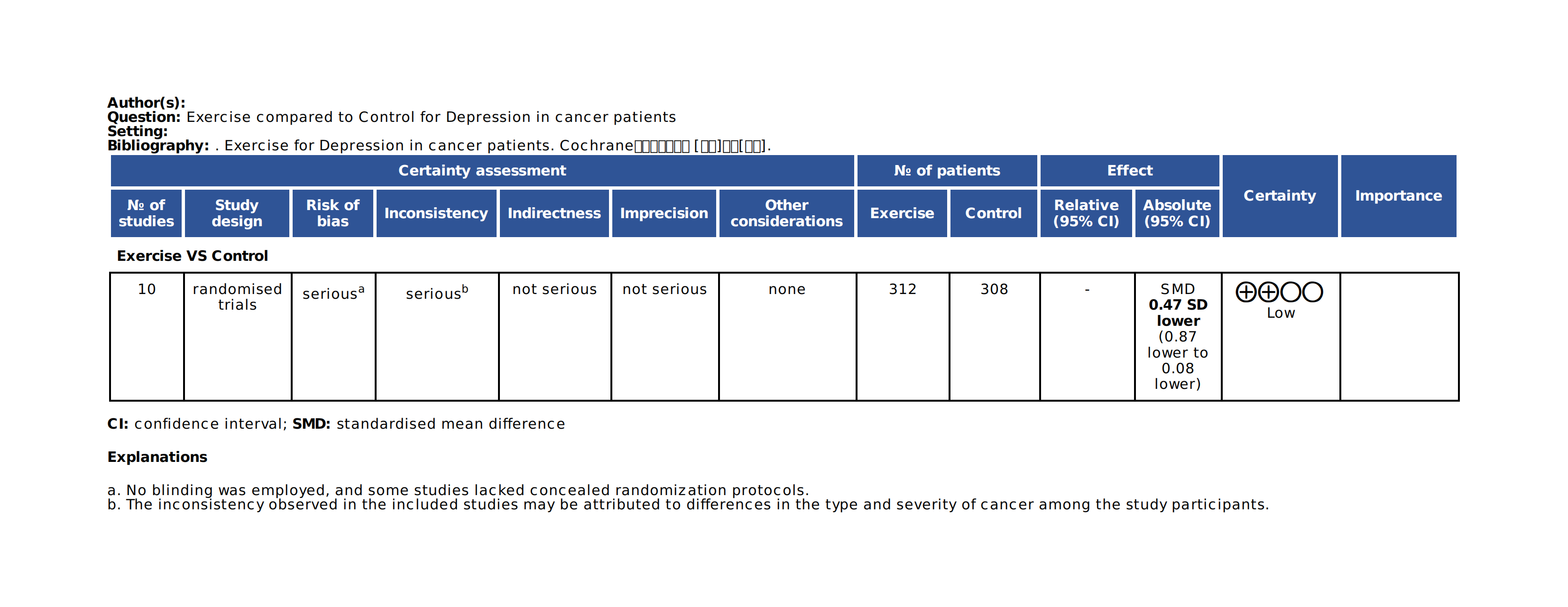

Supplement: Supplementary file 6 — Supplementary Material 6 [file 432_2024_5813_MOESM6_ESM.png]
